# Does dose-dense neoadjuvant chemotherapy have clinically significant prognostic value in breast cancer?: A meta-analysis of 3,724 patients

**DOI:** 10.1371/journal.pone.0234058

**Published:** 2020-05-29

**Authors:** Yuqin Ding, Kaijing Ding, Hongjian Yang, Xiangming He, Wenju Mo, Xiaowen Ding

**Affiliations:** 1 Department of Breast Surgery, Institute of Cancer Research and Basic Medical Sciences of Chinese Academy of Sciences, Cancer Hospital of University of Chinese Academy of Sciences, Zhejiang Cancer Hospital, Hangzhou, Zhejiang, China; 2 Department of Child Psychology, Zhejiang University Affiliated Mental Health Center, Hangzhou Seventh People’s Hospital, Hangzhou, Zhejiang, China; Istituto di Ricovero e Cura a Carattere Scientifico Centro di Riferimento Oncologico della Basilicata, ITALY

## Abstract

**Purpose:**

Neoadjuvant chemotherapy (NCT) is typically the initial treatment for non-early breast cancer patients. We thereby conducted a meta-analysis to explore whether dose-dense neoadjuvant chemotherapy (ddNCT) improved the long-term prognosis of patients compared to the standard NCT regimen.

**Methods:**

We compared the differences in efficacy and prognosis between patients receiving standard NCT and ddNCT. We also calculated the pooled odds ratio (OR) of pathological complete response (pCR) and the pooled hazard ratio (HR) of overall survival (OS) and disease-free survival (DFS).

**Results:**

Nine randomized controlled trials involving 3,724 patients from 10 published studies were included in the meta-analysis. The pooled OR for ddNCT was 1.18 (95% confidence interval (CI): 0.83–1.67, P = 0.356). A subgroup analysis in the cases with low hormone receptor expression levels showed the pCR in patients undergoing ddNCT was significantly higher than the pCR in patients undergoing standard NCT (OR = 1.36, 95% CI: 1.09‒1.69, P = 0.007). There was no significant difference in DFS and OS between ddNCT and standard NCT (DFS: HR = 0.90, 95% CI: 0.79‒1.02, P = 0.095; OS; HR = 0.91, 95% CI: 0.81‒1.04, P = 0.160), regardless of hormone receptor expression levels. These data suggested the higher pCR rate in patients receiving ddNCT did not result in a survival benefit.

**Conclusions:**

The meta-analysis demonstrated that ddNCT can significantly improve the pCR rate in patients with low hormone receptor expression levels, although patient survival was not significantly improved. The ddNCT can increase the breast-conserving rate and reduced pre-operative waiting time without increasing adverse reactions. This regimen can be considered when developing an NCT plan.

## Introduction

The adjuvant anthracycline and taxane-based chemotherapies have been demonstrated to be superior to other regimens and can reduce mortality by about one-third in early breast cancer patients[1, 2]. As an adjuvant treatment, dose-dense chemotherapy is designed to maintain high blood drug concentrations by delivering drugs in a short time, thus achieving maximum tumor-killing effect. In an early phase III prospective randomized clinical trial (CALGB9741), researchers found that dose-dense chemotherapy improved the prognosis (including disease-free survival (DFS) and overall survival (OS) in early breast cancer patients with lymph node metastases. The treatment reduced recurrent risk by 26% and death risk by 31%[3]. The National Comprehensive Cancer Network (NCCN) guideline (2019 version) also recommends the use of anthracycline or taxane-based dose-dense regimens as adjuvant or neoadjuvant chemotherapy in breast cancer[4].

Neoadjuvant chemotherapy (NCT) is a preoperative systemic treatment commonly used in patients with locally advanced breast cancer or large, yet operable, tumors. The purpose of neoadjuvant treatment includes making a previously inoperable locally advanced tumor operable, and improving the conserving aspect of breast surgery. Previous studies have shown that pathological complete response (pCR) may be associated with a favorable prognosis[5–8]. Therefore, achieving a high pCR rate by increasing the intensity of neoadjuvant chemotherapy is of interest in the treatment setting. In a systematic retrospective meta-analysis conducted by the Early Breast Cancer Trialists’ Collaborative Group (EBCTCG) in 2019, it was found that compared with the standard regimen, the 10-year recurrence and mortality rates were significantly reduced in the population receiving dose-dense chemotherapy treatment (P<0.0001)[9]. However, the study did not analyze adjuvant chemotherapy and neoadjuvant chemotherapy separately.

We searched randomized controlled trials (RCTs) including anthracycline or taxane-based dose-dense NCT (ddNCT) regimens. The definition of dose-dense regimens varied within the studies. We systematically reviewed the literature to evaluate the efficacy and prognostic significance of ddNCT for early and locally advanced operable breast cancer.

## Methods

### Search strategy

We searched for literature in the Medline, Web of Science, EMBASE and Cochrane Trials databases using the keywords “dose-dense chemotherapy” or “dose intense” or “every 14 days” or “every week” or “every 2 weeks,” and “breast neoplasms” or “breast cancer” or “breast carcinoma” or “invasive ductal carcinoma (IDC),” and “neoadjuvant therapy” or “preoperative.” The publication time period was included January 1990-October 2019. We also searched the references of these studies and used “similar articles” in PubMed to find as many potential qualified studies as possible. The Preferred Reporting Item Guidelines for Systematic Reviews and Meta-analyses guidelines were followed.

### Inclusion and excluded criteria

Studies meeting the following criteria were included in the present meta-analysis: (1) The diagnosis of breast cancer in the study must be confirmed by histopathological examination (including needle biopsy and lumpectomy biopsy); (2) Patients included in the study were not those who relapsed or had distant metastatic tumors; (3) The definitions of pCR, OS and DFS were clearly stated in the study; (4) Only studies with a score of greater than six in the Newcastle-Ottawa Scale (NOS) were included.

Exclusion criteria included: (1) abstracts, letters, case reports, retrospective studies, and non-clinical trial studies, (2) non-English studies, (3) being given other adjuvant treatments during the dose-dense chemotherapy treatment, and (4) phase II RCTs that only evaluated the safety of dose-dense chemotherapy regimens.

### Data extraction and quality assessment

All candidate publications were independently reviewed and extracted by two authors. For publications that could not be classified according to the title and abstract, the full text was retrieved and reviewed. For each publication, the following items were recorded: first author, publication date, country, the number of cases, follow-up time, treatment regimen, the number of pCR events, OR of pCR, and the hazard ratio (HR) of DFS and OS events. Study quality was assessed based on the NOS[10]. The scale consists of three parts: selection (0–4 points), comparability (0–2 points) and outcome (0–3 points). An NOS score >6 was considered to be a high quality study and was included in the meta-analysis.

### Statistical analysis

The OR, HR and 95% confidence interval (CI) were directly extracted from each publication, or estimated using the method of Parmer et al. An OR >1 indicated a higher pCR rate in the ddNCT group, and an HR <1 indicated a better prognosis in the ddNCT group. Cochran’s Q test and Higgins I-squared analyses were used to assess the heterogeneity of the studies. Fixed effects (Mantel-Haenszel method) or random effects (DerSimonia-Laird method) models were used to calculate the pooled HR and 95% CI. A heterogeneity P value <0.10 or I^2^> 50% indicated that a significant heterogeneity existed. A random effects model was used in the case of heterogeneity. Otherwise, a fixed effects model was used. A subgroup analysis was used to explore and explain the differences (heterogeneity) among various studies. Publication bias was evaluated using Begg’s funnel plot and an Egger’s linear regression test. All P values were two-sided. P<0.05 was considered statistically significant. All statistical analyses were performed using Stata 15.0 (STATA, College Station, TX).

### Ethical approval

This article does not contain any studies with human participants or animals.

## Results

### Study characteristics

**Fig 1:**
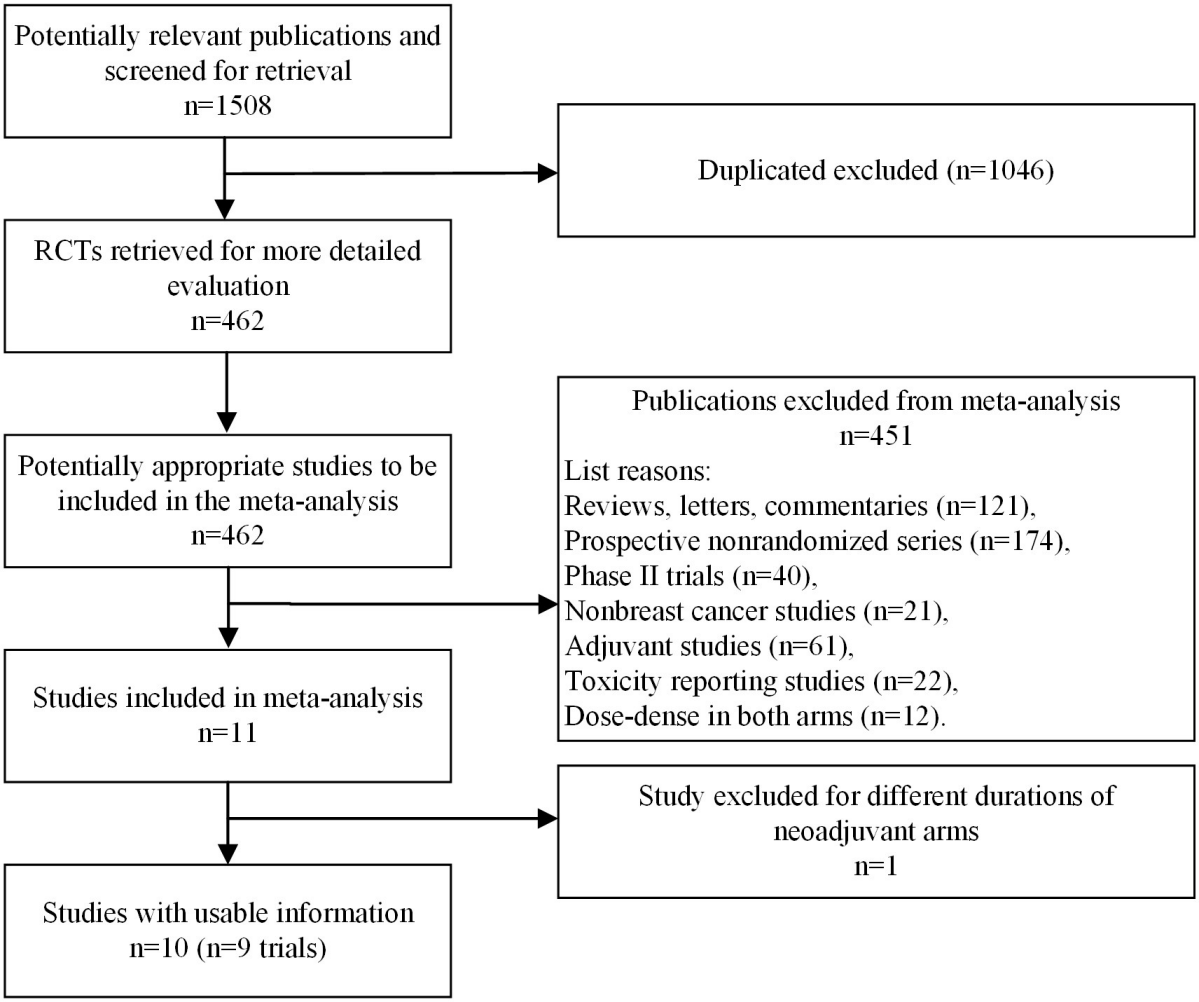
Search process.

The initial literature search resulted in a total of 1,508 publications. After careful review of the publications, nine studies from 10 publications were included in the final meta-analysis[2, 11–19]. A total of 3,724 patients were included, and among them, 1,857 patients received ddNCT and 1,867 patients received standard NCT. The workflow of the literature search and review is summarized in Fig 1. Six studies were conducted in Europe and three studies were conducted in the United States. The OR values of the pCR rate were reported in seven studies. In two studies, OR values were calculated using a univariate analysis of the pCR rate. There were six studies reporting the association between ddNCT and prognosis. Among these studies, five provided the HR and 95% CI, and the HR value in the remaining study was calculated with a multivariate analysis. Three studies had fewer than 200 cases, while six studies had more than 200 cases. One study involved inflammatory breast cancer, and the other eight studies included only patients with stage II-III breast cancer. The characteristics of the included studies are shown in S1 Table. The chemotherapy regimens in all studies are listed in S2 Table.

### ddNCT and pCR analyses

**Fig 2:**
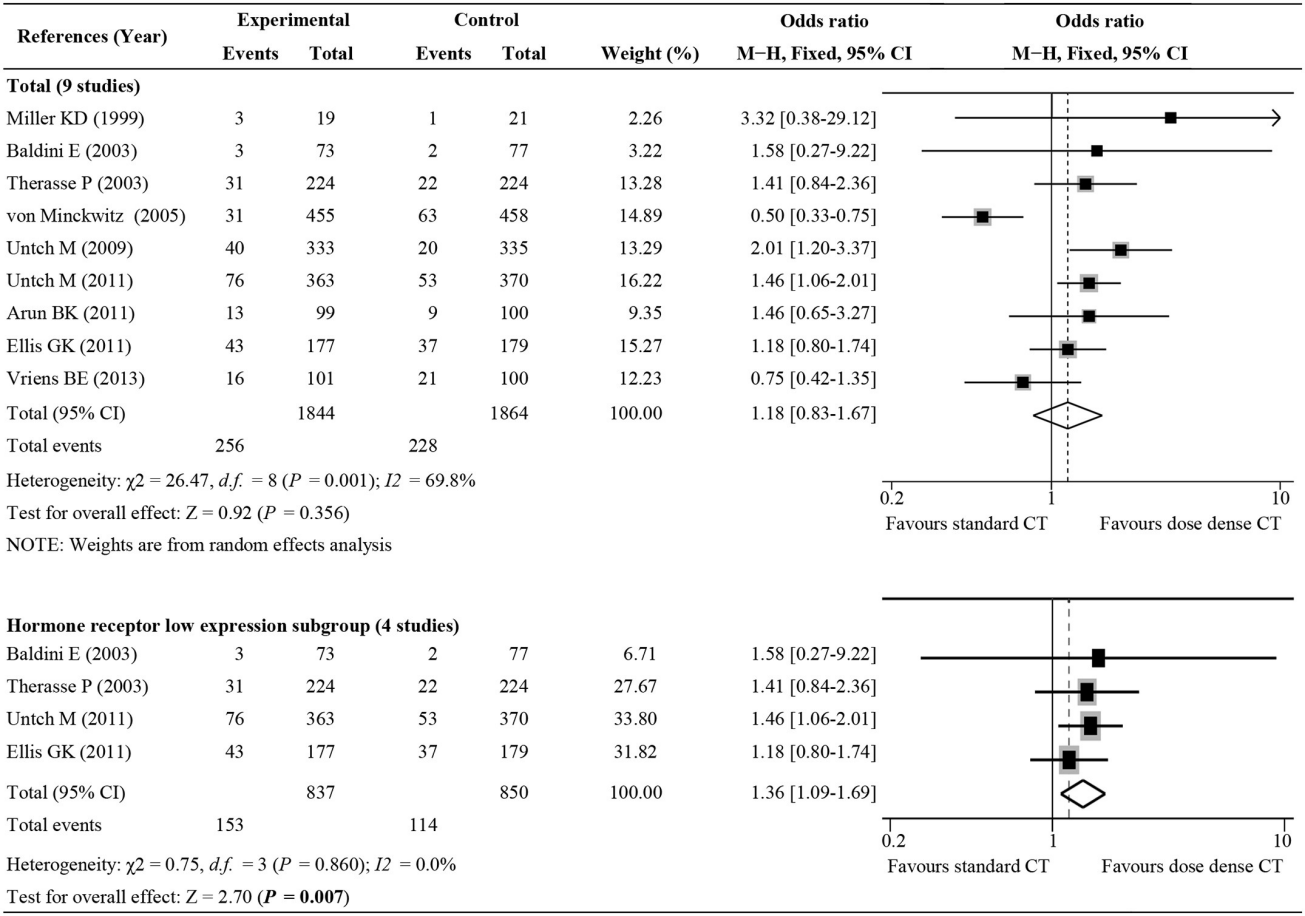
Forest plot of odds ratios (ORs) comparing pathologic complete response for all patients who received dose-dense chemotherapy versus those who received standard chemotherapy. CI, confidence interval.

Nine studies provided the pCR rates of ddNCT and standard NCT. A random effects model was used because of the presence of significant heterogeneity (I^2^ = 69.8%, P_h_ = 0.001). The overall pCR rate was 13.07% (485/3708) and the pooled OR was 1.18 (95% CI: 0.83–1.67, P = 0.356; Fig 2). The pCR rate in the population receiving ddNCT was not significantly improved compared to the pCR rate in the overall population. There were four studies involving patients with low hormone receptor expression levels (more than 50% of patients were estrogen receptor and progesterone receptor negative). Five studies included patients with relatively high hormone receptor expression. We thereby performed a subgroup analysis based on hormone receptor expression level. The improvement of the pCR rate was significant higher in patients with low hormone receptor expression levels and who underwent ddNCT treatment (OR = 1.36, 95% CI: 1.09–1.69, P = 0.007; Fig 2)[2, 11, 15, 17, 19].

### ddNCT, OS, and DFS

**Fig 3:**
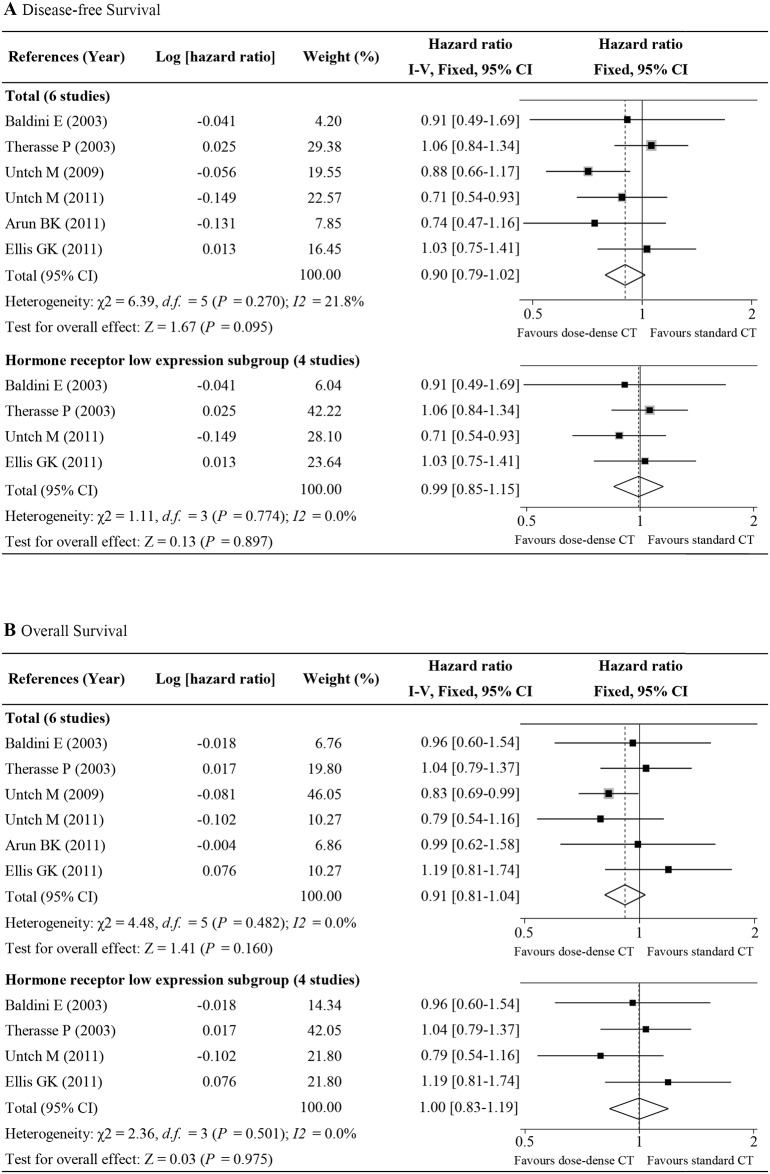
Forest plot of hazard ratios comparing disease-free survival (A) and overall survival (B) for all patients who received dose-dense chemotherapy versus those who received standard chemotherapy. CI, confidence interval.

Six studies with 2,750 patients provided OS and DFS data for breast cancer patients undergoing ddNCT treatment[2, 11, 15–19]. A pooled analysis demonstrated that ddNCT improved patient prognosis but this did not achieve statistical significance (DFS: HR = 0.90, 95% CI: 0.79–1.02, P = 0.095, Fig 3A; OS: HR = 0.91, 95% CI: 0.81–1.04, P = 0.160, Fig 3B). A subgroup analysis based on hormone receptor expression level did not demonstrate a significant improvement in prognosis in the patients with low hormone receptor expression level and who underwent ddNCT treatment (DFS HR = 0.99, 95% CI: 0.85–1.15, P = 0.897, Fig 3A; OS HR = 1.00, 95% CI: 0.83–1.19, P = 0.975, Fig 3B). Six studies did not have significant heterogeneity, so a fixed effects model was used to assess the DFS (I^2^ = 21.8%, P_h_ = 0.270) and OS (I^2^ = 0%, P_h_ = 0.482).

### Publication bias

**Fig 4:**
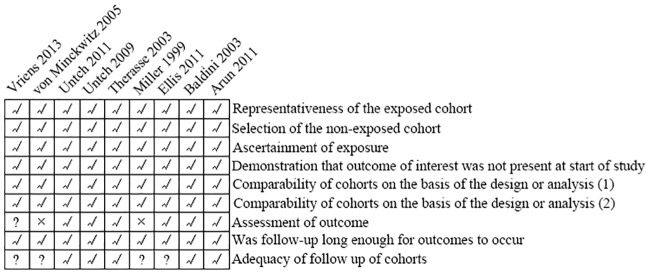
QUADAS-2 risk of bias of the studies included. √ low risk; ? unclear risk; × high risk.

**Fig 5:**
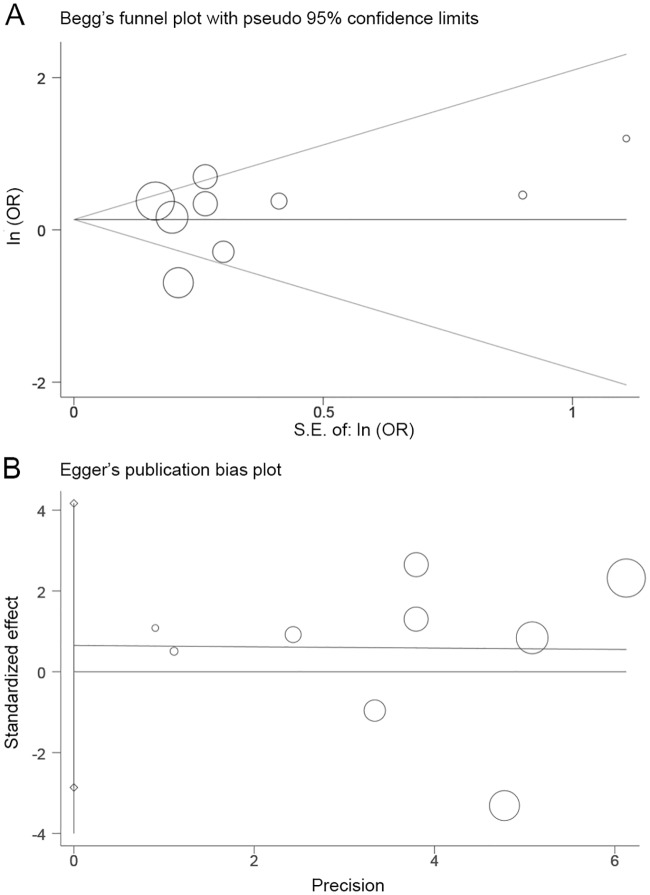
Begg’s funnel plot (A) and Egger’s linear regression test (B) of odds ratio (OR) of pathologic complete response (pCR) in dose-dense arms versus standard arms.

Detailed QUADAS-2 scale scoring is shown in Fig 4. In general, the bias risk was low. We also used Begg’s funnel plots and an Egger’s linear regression test to evaluate publication bias. Significant publication bias was not detected (Pr> |z| = 1.000 for Begg’s test and P> |t| = 0.968 for Egger’s test; Fig 5).

## Discussion

The theory of dose-dense chemotherapy is based on the Norton-Simon hypothesis that posits that a large proportion of tumor cells are in a resting phase (G0 phase) rather than a proliferative phase[20]. These quiescent cells are not sensitive to high-dose chemotherapy. Dose-dense chemotherapy can shorten the interval between doses, therefore allowing tumor cells to be exposed to cytotoxic drugs more frequently. This affects growth signals in tumor cells to a greater extent and achieves maximum tumor cell-killing effects. Our present meta-analysis showed that compared with patients receiving standard NCT, patients receiving ddNCT had a higher pCR rate (increased by 18%). However, the increase in the pCR was not statistically significant in the overall population. We performed a subgroup analysis for the four studies that included patients with low hormone receptor expression levels. We found that in this subgroup, patients obtained a significantly higher pCR rate (increased by 35%) when they received ddNCT. This finding was consistent with previous studies[21, 22]. Therefore, ddNCT may benefit patients with low hormone receptor expression levels or with triple-negative breast cancer by increasing pCR rates and improve downstage and breast-conserving rates. Such a clinical benefit was not observed in the broader population. Therefore, the target population for ddNCT should be carefully determined. Unfortunately, because most included studies in our meta-analysis did not provide detailed information of patient characteristics, we could not perform further subgroup analyses for patients with certain high-risk factors (ex. young age, multiple lymph node metastases, large tumor size, triple-negative breast cancer). In fact, prior studies suggest these patients have a greater chance of benefiting from ddNCT treatment[23].

The NSABP-B27 trial suggests that for operable breast cancer patients with a high tumor burden, the dose-dense regimen of doxorubicin in combination with cyclophosphamide followed by docetaxel sequential therapy could increase the pCR rate of ddNCT from 13% to 27%[21]. Such a benefit may ultimately translate into an improvement of survival. The phase III MA21 trial demonstrated that the dose-dense EC-T regimen (epirubicin/cyclophosphamide followed by paclitaxel) is superior to the standard three-week AC-T regimen (doxorubicin/cyclophosphamide followed by paclitaxel) in improving DFS for high-risk operable patients. This regimen is safer than the CEF regimen (cyclophosphamide, epirubicin, and fluorouracil), with comparable efficacy[24]. Our analysis demonstrated that for the overall population, ddNCT resulted in a survival benefit. ddNCT therapy decreased the recurrence risk by about 10% and the mortality risk by about 8%, which were close to statistical significance. However, we did not find an enhanced benefit in the low hormone receptor expression level subgroup. The pCR rate did increase in this subgroup when ddNCT was given, indicating that the pCR rate is not a good surrogate for survival outcomes. Our results were consistent with the results from the Berruti study[25]. The included studies in our meta-analysis had long study times. Moreover, the information on postoperative adjuvant treatments was not specified in most studies. The ddNCT-associated survival benefit may be attenuated or masked in some early-stage patients who receive standardized surgery and postoperative adjuvant treatments. In addition, Her2 status was not provided in some studies, therefore, the effect of Her2 status on ddNCT efficacy could not be investigated. For Her2-positive patients, ddNCT may further improve patient pCR rates and prognosis when combined with trastuzumab targeted therapy.

Although prognoses improved, the primary endpoint of the present study did not reach statistical significance in the general population. However, ddNCT still demonstrated certain advantages over the standard NCT. For breast cancer patients with a high tumor burden or locally advanced tumors that preclude surgical resection, ddNCT can shorten total treatment time and patients can undergo surgery after ddNCT. Additionally, a higher pCR rate may result in more patients having the opportunity to preserve breast tissue and have an improved quality of life. Previous studies have confirmed that dose-dense chemotherapy regimens are more effective for certain types of breast cancer, such as triple-negative breast cancer[26, 27]. Although we did not systematically evaluate the safety of ddNCT treatments, we found that the majority of patient were tolerant of the treatment and completed the treatment regimens. Although hematological toxicities are more likely to occur in the ddNCT group, the use of recombinant human granulocyte colony-stimulating factor can largely prevent serious hematological adverse reactions.

There were several limitations in this retrospective meta-analysis. The definition of “dose dense” in the nine RCT studies from 10 publications varied, leading to a bias in evaluating treatment outcomes. The limited number of included studies restricted the power of the analysis in the evaluation of the prognostic value of ddNCT. When the number of the included studies is less than 10, the power of Egger’s and Begg’s testing is low and may not detect publication bias efficiently. In addition, only English publications were included in the study. Therefore, publication bias could not be completely ruled out. Lastly, heterogeneity among the studies may also affect the interpretation of results. Heterogeneity may be introduced by multiple factors such as patient age, tumor size, molecular subtyping, and lymph node metastasis status.

In conclusion, our meta-analysis suggested that ddNCT can increase the pCR rate, especially for those with low hormone receptor expression. In the general population, ddNCT can improve DFS and OS, but this benefit did not achieve statistical significance. Therefore, large sample prospective studies are needed to validate our findings.

## Supporting information

S1 Table Characteristics of the studies included. * Dose-dense arm. Abbreviations: A = doxorubicin; C = cyclophosphamide; DOC = docetaxel; E = epirubicin; ER = estrogen receptor; F = fluorouracil; M = methotrexate; NR = not reported; P = paclitaxel; pCR = pathological complete response; PR = progesterone receptor; T = docetaxel.
(PDF)

S2 Table Trials chemotherapy protocols. Abbreviations: A = doxorubicin; C = cyclophosphamide; DOC = docetaxel; E = epirubicin; F = fluorouracil; M = methotrexate; P = paclitaxel.
(PDF)

S1 File (DOC)

S2 File (PDF)

S1 Dataset (XLS)
