# Supplementary material for: Does dose-dense neoadjuvant chemotherapy have clinically significant prognostic value in breast cancer?: A meta-analysis of 3,724 patients
Source: PLoS One. 2020 May 29;15(5):e0234058. doi: 10.1371/journal.pone.0234058 (PMC7259732; doi:10.1371/journal.pone.0234058)
Supplement: S1 Table — * Dose-dense arm. Abbreviations: A = doxorubicin; C = cyclophosphamide; DOC = docetaxel; E = epirubicin; ER = estrogen receptor; F = fluorouracil; M = methotrexate; NR = not reported; P = paclitaxel; pCR = pathological complete response; PR = progesterone receptor; T = docetaxel. (PDF) [file pone.0234058.s001.pdf]

Table 1. Characteristics of the studies included

First author, year (reference)	Trial name	Study location	Treatment protocol	Treatment interval, days	Number of patients	Median follow-up, months	Median age (range)	Stage		ER positive , %	PR positive , %	pCR events (%)
								Tumor size, %	Nodal status, %			
Baldini E, 2003 (11)	PisaGenoa	Italy	*CEF × 3 - surgery radiation - CMF and CEF intercalated × 6	14	77	60	52 (30–70)	IIIA: 22.7, IIIB: 49.3, IBC: 24		43	34	3 (4.1)
			CEF × 3 - surgery radiation - CMF and CEF intercalated × 6	21	73		50 (30–70)			38.4	26	2 (2.6)
Therasse P, 2003 (15)	EORTC-NCIC-SAKK	Europe	*EC × 6	14	224	66	49 (16-72)	T0-2: 4, T3: 9, T4: 86	N0-1: 58, N2-3: 40	42	NR	31 (14.0)
			CEF × 6	28	224		49 (29-79)	T0-2: 4, T3: 9, T4: 86	N0-1: 54, N2-3: 44	38	NR	22 (10.0)
von Minckwitz G, 2005 (13	GeparDuo	Germany	*A DOC × 4	14	455	60	52 (24–77)	T1: 6; T2: 85.4; T3: 8.7	Positive 37.6	65.3	61.9	31 (7.0)
			AC × 4 - DOC × 4	21	458		51 (24–74)	T1: 8; T2: 84.4; T3: 7.7	Positive 49.9	69.5	55.1	63 (14.3)
Untch M, 2011 (2, 19)	PREPARE	Germany	*ddE × 3 - ddP × 3 - CMF × 3	14	363	62.4	48 (23–65)	T1–3: 86.2,T4: 8.5	Positive 37.2	ER and/or PR positive 41.6		76 (20.9)
			EC × 4 - P × 4	21	370		49 (26–65)	T1–3: 90.8,T4: 7.3	Positive 38.4	ER and/or PR positive 43		53 (14.3)
Vriens BE, 2013 (14)	BOOG	Netherlands	*TAC × 6	21	101	NR	49 (24–68)	T1: 5, T2: 45, T3: 30, T4: 20	Positive 74	ER and/or PR positive 65		16 (16.0)
			AC × 3 - T × 3	21	100		49 (27–70)	T1: 2, T2: 49, T3: 34, T4: 15	Positive 76	ER and/or PR positive 66		21 (21.0)
Miller KD, 1999 (12)	Hoosier	America	*A × 3 - DOC × 3	14	19	NR	47 (31-60)	stage II or noninflammatory stage III: 100		NR		3 (15.7)
			A DOC × 4	21	21		46 (28-63)			NR		1 (4.7)
Untch M, 2009 (16)	AGO-1	Germany	*E × 3 - P × 3	14	333	54	49 (27-68)	T2: 55, T3: 27,T4: 18	Positive 54	Hormone receptor positive: 66		40 (12.0)
			EP × 4	21	335		51 (26-66)	T2: 51, T3: 31, T4: 18	Positive 54	Hormone receptor positive: 69		20 (6.0)
Arun BK, 2011 (18)	Houston	America	*FAC × 4	18	99	91.5	NR	T0: 1, T1: 4, T2: 23, T3: 22, T4: 50	Positive 88.2	39.5	39.5	13 (13.1)
			FAC × 4	21	100		NR	T0: 2, T1: 7.8, T2: 18.6, T3: 22.5, T4: 4	Positive 90	47.1	55.3	9 (9.0)
Ellis GK, 2011 (17)	SWOG-0012	America	*wAC × 12-wP × 12	7	186	60	52 (27-76)	IIB: 12, IIIA 31, IIIB 52		Hormone receptor positive: 55		43 (24.3)
			AC × 4 - wP × 12	21	186		51 (22-77)	IIB: 19, IIIA 31, IIIB 48		Hormone receptor positive: 46		37 (20.7)

* Dose-dense arm.

Abbreviations: A = doxorubicin; C = cyclophosphamide; DOC = docetaxel; E = epirubicin; ER = estrogen receptor; F = fluorouracil; M = methotrexate; NR = not reported; P = paclitaxel; pCR = pathological complete response;PR = progesterone
