# Supplementary material for: Does dose-dense neoadjuvant chemotherapy have clinically significant prognostic value in breast cancer?: A meta-analysis of 3,724 patients
Source: PLoS One. 2020 May 29;15(5):e0234058. doi: 10.1371/journal.pone.0234058 (PMC7259732; doi:10.1371/journal.pone.0234058)
Supplement: S2 Table — Abbreviations: A = doxorubicin; C = cyclophosphamide; DOC = docetaxel; E = epirubicin; F = fluorouracil; M = methotrexate; P = paclitaxel. (PDF) [file pone.0234058.s002.pdf]

Table 2. Trials chemotherapy protocols.

Study	Conventional arm	Dose-dense arm
Baldini E, 2003 (11)	C: 600 mg/m2 + E: 60 mg/m2 + F: 600 mg/m2, every 3 weeks for 3 cycles then local therapy then CEF (same) intercalated by C: 600 mg/m2 + M: 60 mg/m2 + F: 600 mg/m2 every 3 weeks for a total of 6	C: 600 mg/m2, E: 60 mg/m2, F: 600 mg/m2, every 2 weeks for 3 cycles then local therapy then CEF (same) intercalated by C: 600 mg/m2 + M: 60 mg/m2 + F: 600 mg/m2 every 2 weeks for a total of 6 cycles
Therasse P, 2003 (15)	C: 75 mg/m2 (orally days 1 to 14) + E: 60 mg/m2 (days 1 and 8) + F: 500 mg/m2 (days 1 and 8), every 28 days for 6 cycles	E: 120 mg/m2 + C: 830 mg/m2, every 14 days for 6 cycles
von Minckwitz G, 2005 (13)	A: 60 mg/m2 + C: 600 mg/m2 4 cycles , then DOC: 75 mg/m2, every 3 weeks for 4 cycles	A: 50 mg/m2 + DOC: 75 mg/m2, every 2 weeks for 4 cycles
Untch M, 2011 (2, 19)	E: 90 mg/m2 + C: 600 mg/m2 every 3 weeks for four cycles then P: 175 mg/m2 every 3 weeks for 4 cycles	E: 150 mg/m2 every 2 weeks for 3 cycles then P: 225 mg/m2 every 2 weeks for 3 cycles then CMF (C: 500 mg/m2, M: 40 mg/m2, and F: 600 mg/m2) , days 1 and 8, every 4 weeks for 3 cycles
Vriens BE, 2013 (14)	A: 60 mg/m2 + C: 600 mg/m2, every 3 weeks for 4 cycles then DOC: 100 mg/m2 , every 3 weeks for 4 cycles	A: 75 mg/m2 + C: 500 mg/m2 + DOC: 50 mg/m2 , every 3 weeks for 6 cycles
Miller KD, 1999 (12)	A: 75mg/m2 every 2 weeks for 3 cycles then DOC: 100 mg/m2 every 2 weeks for 3 cycles	A: 56 mg/m2 + DOC: 75 mg/m2 every 3 weeks for 4 cycles
Untch M, 2009 (16)	E: 90 mg/m2 + P: 175 mg/m2 every 3 weeks for 4 cycles	E: 150 mg/m2, every 14 days for 3 cycles then P: 250 mg/m2, every 14 days for 3 cycles
Arun BK, 2011 (18)	F: 500 mg/m2 + A: 50 mg/m2 + C: 500 mg/m2, every 21 days for 4	F: 600 mg/m2 + A: 60 mg/m2 + C: 1000 mg/m2, every 18 days for 4
Ellis GK, 2011 (17)	A: 60 mg/m2 + C: 600 mg/m2, every 3 weeks for 5 cycles, then local therapy, then P: 80 mg/m2/wk for 12 weeks	A: 24 mg/m2/wk, every week for 15 weeks + C: 60 mg/m2/d, orally each day for 15 weeks, then local therapy, then P: 80 mg/m2/wk for 12

Abbreviations: A = doxorubicin; C = cyclophosphamide; DOC = docetaxel; E = epirubicin; F = fluorouracil; M = methotrexate; P = paclitaxel.
