# Supplementary material for: Does dose-dense neoadjuvant chemotherapy have clinically significant prognostic value in breast cancer?: A meta-analysis of 3,724 patients
Source: PLoS One. 2020 May 29;15(5):e0234058. doi: 10.1371/journal.pone.0234058 (PMC7259732; doi:10.1371/journal.pone.0234058)
Supplement: S2 File — (PDF) [file pone.0234058.s004.pdf]

PubMed MEDLINE

Search	Query	Items found	Time
#1	Search **"Breast Neoplasms"[Mesh]** Sort by: **Best Match**	288560	22:52:08
#2	Search ((((((((((Breast Neoplasm[Title/Abstract]) OR Breast Tumor[Title/Abstract]) OR Breast Cancer[Title/Abstract]) OR Mammary Cancer[Title/Abstract]) OR Malignant Neoplasm of Breast[Title/Abstract]) OR Breast Malignant Neoplasm[Title/Abstract]) OR Malignant Tumor of Breast[Title/Abstract]) OR Breast Malignant Tumor[Title/Abstract]) OR Breast Malignant Tumors[Title/Abstract]) OR Cancer of Breast[Title/Abstract]) OR Cancer of the Breast[Title/Abstract]) OR Breast Carcinoma[Title/Abstract] Sort by: Best Match	288418	22:54:35
#3	Search ((((((((((((Breast Neoplasm[Title/Abstract]) OR Breast Tumor[Title/Abstract]) OR Breast Cancer[Title/Abstract]) OR Mammary Cancer[Title/Abstract]) OR Malignant Neoplasm of Breast[Title/Abstract]) OR Breast Malignant Neoplasm[Title/Abstract]) OR Malignant Tumor of Breast[Title/Abstract]) OR Breast Malignant Tumor[Title/Abstract]) OR Breast Malignant Tumors[Title/Abstract]) OR Cancer of Breast[Title/Abstract]) OR Cancer of the Breast[Title/Abstract]) OR Breast Carcinoma[Title/Abstract])) AND "Breast Neoplasms"[Mesh] Sort by: Best Match	206186	22:55:16
#4	Search "Neoadjuvant Therapy"[Mesh] Sort by: Best Match	20055	22:56:51
#5	Search ((Neoadjuvant Therapies[Title/Abstract]) OR Neoadjuvant Treatment[Title/Abstract]) OR Neoadjuvant Treatments[Title/Abstract]	3682	22:58:45
#6	Search ((((Neoadjuvant Therapies[Title/Abstract]) OR Neoadjuvant Treatment[Title/Abstract]) OR Neoadjuvant Treatments[Title/Abstract])) OR "Neoadjuvant Therapy"[Mesh] Sort by: Best Match	22134	22:59:03
#7	Search chemotherapy[Title/Abstract]	359045	23:01:11
#8	Search ((((((Neoadjuvant Therapies[Title/Abstract]) OR Neoadjuvant Treatment[Title/Abstract]) OR Neoadjuvant	12262	23:01:31

	Treatments[Title/Abstract])) OR "Neoadjuvant Therapy"[Mesh])) AND chemotherapy[Title/Abstract] Sort by: Best Match		
#9	Search (dose) AND dense Sort by: Best Match	3190	23:02:27
#10	Search (dose) AND intense	5323	23:02:52
#11	Search (every) AND 14	40944	23:03:07
#12	Search ((every) AND 14) AND days	13535	23:03:30
#13	Search (every) AND week	24788	23:03:55
#14	Search (((((dose) AND dense)) OR ((dose) AND intense)) OR (((every) AND 14) AND days)) OR ((every) AND week)	44796	23:04:26
#15	Search ((((((((dose) AND dense)) OR ((dose) AND intense)) OR (((every) AND 14) AND days)) OR ((every) AND week))) AND (((((((Neoadjuvant Therapies[Title/Abstract]) OR Neoadjuvant Treatment[Title/Abstract]) OR Neoadjuvant Treatments[Title/Abstract])) OR "Neoadjuvant Therapy"[Mesh])) AND chemotherapy[Title/Abstract])) AND ((((((((((((((Breast Neoplasm[Title/Abstract]) OR Breast Tumor[Title/Abstract]) OR Breast Cancer[Title/Abstract]) OR Mammary Cancer[Title/Abstract]) OR Malignant Neoplasm of Breast[Title/Abstract]) OR Breast Malignant Neoplasm[Title/Abstract]) OR Malignant Tumor of Breast[Title/Abstract]) OR Breast Malignant Tumor[Title/Abstract]) OR Breast Malignant Tumors[Title/Abstract]) OR Cancer of Breast[Title/Abstract]) OR Cancer of the Breast[Title/Abstract]) OR Breast Carcinoma[Title/Abstract])) AND "Breast Neoplasms"[Mesh])	111	23:05:49
